# Supplementary material for: The effect of notification window length on the epidemiological impact of COVID-19 contact tracing mobile applications
Source: Commun Med (Lond). 2022 Jun 27;2:74. doi: 10.1038/s43856-022-00143-2 (PMC9237034; doi:10.1038/s43856-022-00143-2)
Supplement: Supplementary file 1 — Supplementary Material [file 43856_2022_143_MOESM1_ESM.pdf]

Supplementary Information: The effect of notification window length on the epidemiological impact of COVID-19 contact tracing mobile applications

Trystan Leng[1,2,3*], Edward M. Hill[1,2], Matt J. Keeling[1,2], Michael J. Tildesley[1,2†], Robin N. Thompson[1,2†]

1. The Zeeman Institute for Systems Biology & Infectious Disease Epidemiology Research, School of Life Sciences and Mathematics Institute, University of Warwick, Coventry, United Kingdom

2. JUNIPER – Joint UNIversities Pandemic and Epidemiological Research, https://maths.org/juniper/

3. Department of Infectious Disease Epidemiology, Imperial College London, London, United Kingdom

†These authors contributed equally to this work.

*Corresponding Author. Email: trystan.leng@imperial.ac.uk

Supplementary Methods

In this analysis, we consider a base case asymptomatic app user infected on day 0 who tests positive and self-isolates from day d. The relative probability that a base case individual is detected on day d depends on the likelihood that they test positive upon taking a test. We define $T(d)$ as the probability density function of the time at which a base case is detected. We obtain this using a test probability profile for lateral flow tests for asymptomatic testing[1], assuming that detected cases are sampled according to their probability of testing positive that day. By considering the expected number of primary cases that are infected by a base case individual, the expected number of secondary cases that result from those primary cases, and by varying the probability p that notified primary cases are active app users who adhere to their notification and consequently self-isolate, we can understand the reduction in transmission that results from different levels of active app use for different notification windows.

The expected number of infections resulting from an infected individual depends on an individual's infectivity profile through time since becoming infected, as well as on the time periods when an individual is not isolating. We make the simplifying assumption that, assuming an individual is not isolating, their contact rate is constant through time. We let R denote the expected number of secondary infections from a symptomatic individual in a completely unvaccinated population, assuming that a symptomatic individual mixes within the population with no interventions placed upon them for the entirety of their infectious period. While R is a function of an individual's contact rate, we find that R^* under different length notification windows is a linear function of R. Consequently, the relative effectiveness of different notification windows is independent of R, i.e. is independent of the contact rate assumed. Letting $f(t)$ be the probability density function of an infected individual's infectivity profile through time, we set $f = \Gamma(5.62, 0.98)$, corresponding to a previously derived infectivity profile obtained using data from known infector–infectee pairs[2]. We let $F(d)$ represent the cumulative density function of the infectivity profile i.e. $F(d) = \int_0^d f(t)dt$.

We assume that a proportion V of the population are vaccinated, while $U = 1 - V$ remain unvaccinated. We assume that vaccination reduces an individual's susceptibility to infection; only a fraction of c_1 high-risk contacts who are vaccinated will become infected. We also assume that vaccination reduces an individual's transmissibility upon infection by a factor c_2. We set $c_1 = 0.37$, and $c_2 = 0.37$ in line with contemporary estimates of SARS-CoV-2 vaccine efficacy[3,4], and set $V = 0.7$, in line with the proportion of the eligible population to have received two doses of vaccine in the UK on the 2nd August 2021[5]. Accordingly, the expected number of secondary infections from a symptomatic individual with no interventions placed upon them for the entirety of their infectious period is given by $(U + c_1V)R$. In line with guidance to contacted individuals as of August 2021, we assumed that vaccinated primary cases do not have to isolate upon notification. We assume the likelihood of displaying symptoms and distribution of time to symptom onset is unimpacted by vaccination, though in reality these may be affected.

Assuming the base case individual tests positive and inputs their result to their app, all contacts of the base case from time $d - w$ to d are notified of possible exposure, where w denotes the notification window length (in days). Our analysis focuses on the cases $w = 5$ and $w = 2$, the notification window lengths used in the UK prior to and after the 2nd August 2021 respectively [6]. Adhering notified individuals isolate for i_{notif} days upon being notified, counted from the time of last contact. We assume that the time of last contact between the base case and primary case individual was the time the primary case individual was infected by the base case. Notified individuals are expected to isolate for 10 **full** days from the day of last contact [7]. Under this rule, the average isolation period would be expected to be 10.5 days, assuming it is equally likely that the time of last contact is any time of the day. Accordingly, we approximate this by setting $i_{notif} = 10.5$.

We let l denote the delay between the base case taking a test and receiving a result. We assume the base case takes a lateral flow test (LFT) and set $l = 0$. In Supplementary Note 1, we consider an instance where the base case individual takes a PCR test, assuming $l = 2$.

The period an infected individual isolates for will depend on whether they develop symptoms. We assume that a proportion A of primary cases are asymptomatic. Here we set $A = 0.3$, in line with estimates of the proportion of SARS-CoV-2 infections that are asymptomatic reported in a meta-analysis [8]. We assume the same infectivity profile for symptomatic and asymptomatic individuals, but assume that asymptomatic individuals are relatively less infectious by a factor $\alpha, 0 < \alpha \leq 1$. We used $\alpha = 0.5$, in line with previous modelling studies, though estimates obtained from empirical studies vary substantially [9]. Upon symptom onset, we assume that individuals test positive and isolate for a period of i_{sympt} days. In the UK, symptomatic individuals are expected to isolate for 10 full days from symptom onset [10]. We approximate isolating for 10 full days by setting $i_{sympt} = 10.5$. Letting $s(t)$ be the probability density function of the time to symptom onset from day of infection for symptomatic individuals, we set $s(t) = \Gamma(5.807, 0.948)$ [11]. We make the assumption that $s(t)$ and $f(t)$ are independent functions of time - while this is a simplifying assumption, it is one common in the literature [12,13].

A proportion p of the primary cases are assumed to be active app users. Active app users are defined as individuals who both a) have and use the app and b) adhere to isolation upon notification. In our analyses we vary p from 0 to 1.

Below, we consider the expected number of primary cases and secondary cases in turn.

Primary cases

If a base case takes a test on day d and isolates accordingly from day d, then the expected number of infections from the base case prior to isolation is given by:

$$N_P(d) = \alpha R(U + c_1 V) \times F(d) \tag{1}$$

The expected number of infections from a typical base case prior to isolation is given by integrating over the day a base case takes a test, and accounting for the relative probability an individual tests positive that day:

$$N_P = \int_0^\infty T(t)N_P(t)dt = \alpha R(U + c_1 V) \times \int_0^\infty T(t)F(t)dt \tag{2}$$

Secondary cases

Secondary cases infected by unvaccinated primary cases can occur in three different ways, dependent on whether the primary case is infected within the notification window, and whether the primary case is an active app user. In contrast, as vaccinated primary cases are not required to isolate upon notification, the expected number of secondary cases from a vaccinated primary case is not affected by the time at which the primary case was infected. Hence there are four instances to consider, which are considered in turn below:

1. **Secondary cases resulting from unvaccinated primary cases infected before the notification window**

   All unvaccinated primary cases infected by the base case from day 0 to day $d - w$ are not notified of exposure. Asymptomatic individuals remain infectious throughout their infectious period, while symptomatic individuals remain infectious until symptom onset before isolating for i_{sympt} days. For a base case detected on day d, the expected number of secondary infections in this instance is given by:

$$N_{S1}(d) = \alpha RU \times F(d-w) \times R(U + c_1 V)G \tag{3}$$

$$\text{where } G = A\alpha + (1-A)\int_0^\infty [F(\tau) + (1 - F(\tau + i_{sympt})]\, s(\tau)d\tau \tag{4}$$

The expected number of secondary infections in this instance from a typical base case is given by:

$$N_{S1} = \int_0^\infty T(t)N_{S1}(t)dt = \alpha RU \times R(U + c_1 V)G \times \int_0^\infty T(t)F(t-w)dt \tag{5}$$

2. **Secondary cases resulting from unvaccinated primary cases infected within the notification window, but are not active app users**

   A proportion $(1-p)$ of unvaccinated cases are not active app users, and therefore either do not receive a notification, or do not adhere to isolation upon notification. Asymptomatic individuals who are not active app users remain infectious throughout their infectious period, while symptomatic individuals who are not active app users remain infectious until symptom onset, then isolate for i_{sympt} days. Hence, for a base case detected on day d, the expected number of secondary infections in this instance is given by:

$$N_{S2}(d) = \alpha RU \times [F(d) - F(d-w)] \times (1-p)R(U + c_1 V) \times G \tag{6}$$

   The expected number of secondary infections in this instance from a typical base case is given by:

$$N_{S2} = \int_0^\infty T(t)N_{S2}(t)dt = \alpha RU \times (1-p)R(U + c_1 V)G \times \int_0^\infty T(t)[F(t) - F(t-w)]dt \tag{7}$$

3. **Secondary cases resulting from unvaccinated primary cases infected within the notification window who are active app users**

   A proportion Up of primary cases infected from day $d-w$ to d are unvaccinated active app users. These primary cases are notified of possible exposure and adhere to isolation upon notification. They remain infectious until day $d+l$, and could be anywhere from day l to day $w+l$ of their infectious period. In this instance, there are four subcases:

   Subcase a ($N_{S3a}(d)$): Asymptomatic individuals isolate from day $d+l$, and stop isolating i_{notif} full days from their last day of contact with the base case.

$$N_{S3a}(d) = \alpha RU \times pA\alpha R(U + c_1 V)\int_{d-w}^{d} [F(d+l-t) + (1 - F(i_{notif}))]f(t)dt \tag{8}$$

   Subcase b ($N_{S3b}(d)$): Symptomatic individuals who exhibit symptoms before day $d+l$, i.e. before their notification. These individuals are assumed to isolate for i_{sympt} full days from symptom onset, with this isolation period not extended by their subsequent close contact notification.

$$N_{S3b}(d) = \alpha RU \times p(1-A)R(U + c_1 V)\int_{d-w}^{d} f(t)\left(\int_0^{d-t+l} [F(\tau) + (1 - F(\tau + i_{sympt}))]s(\tau)d\tau\right)dt \tag{9}$$

   Subcase c ($N_{S3c}(d)$): Symptomatic individuals infected on day t who exhibit symptoms after day $d+l$ but before day $t + i_{notif}$, i.e. while they are isolating from their notification. These individuals isolate from $d+l$ until i_{sympt} days after the onset of their symptoms.

$$N_{S3c}(d) = \alpha RU \times p(1-A)R(U + c_1 V)\int_{d-w}^{d} f(t)\left(\int_{d-t+l}^{i_{notif}} [F(d+l-t) + (1 - F(\tau + i_{sympt}))]s(\tau)d\tau\right)dt \tag{10}$$

   Subcase d ($N_{S3d}(d)$): Symptomatic individuals infected on day t who exhibit symptoms after $t + i_{notif}$, i.e. after they have finished isolation from their notification. These individuals isolate from $d+l$ until

$t + i_{notif}$, and then leave isolation before isolating once again upon symptom onset, for i_{sympt} days from symptom onset.

$$N_{S3d}(d) = \alpha RU \times p(1-A)R(U+c_1V)\int_{d-w}^{d} f(t)\left(\int_{i_{notif}}^{\infty}[F(d+l-t)+(F(\tau)-F(i_{notif}))+(1-F(\tau+i_{sympt}))]s(\tau)d\tau\right)dt \tag{11}$$

Together, we obtain

$$N_{S3}(d) = N_{S3a}(d)+N_{S3b}(d)+N_{S3c}(d)+N_{S3d}(d) = \alpha RU \times pR(U+c_1V)H(d,w) \tag{12}$$

$$\begin{aligned}\text{where } H(d,w) =& A\alpha\left(\int_{d-w}^{d}[F(d+l-t)+(1-F(i_{notif}))]f(t)dt\right) \qquad (13)\\ &+(1-A)\left\{\int_{d-w}^{d} f(t)\left[\left(\int_{0}^{d-t+l}[F(\tau)+(1-F(\tau+i_{sympt}))]s(\tau)d\tau\right)\right.\right.\\ &+\left(\int_{d-t+l}^{i_{notif}}[F(d+l-t)+(1-F(\tau+i_{sympt}))]s(\tau)d\tau\right)\\ &\left.\left.+\left(\int_{i_{notif}}^{\infty}[F(d+l-t)+(F(\tau)-F(i_{notif}))+(1-F(\tau+i_{sympt}))]s(\tau)d\tau\right)\right]dt\right\}\end{aligned}$$

The expected number of secondary infections in this instance from a typical base case is given by:

$$N_{S3} = \int_0^{\infty} T(t)N_{S3}(t)dt = \alpha RU \times pR(U+c_1V)\int_0^{\infty}T(t)H(t,w)dt \tag{14}$$

4. **Secondary cases resulting from vaccinated primary cases.**

   Vaccinated primary cases are not expected to isolate upon notification, irrespective of whether they are active app users or not, and irrespective of when they were infected. Asymptomatic vaccinated individuals remain infectious throughout their infectious period, while symptomatic vaccinated individuals remain infectious until symptom onset, then isolate for i_{sympt} days. Hence, for a base case detected on day d, the expected number of secondary infections in this instance is given by:

$$N_{S4}(d) = \alpha Rc_1V \times F(d) \times Rc_2(U+c_1V)\times G \tag{15}$$

   The expected number of secondary infections in this instance from a typical base case is given by:

$$N_{S4} = \int_0^{\infty}T(t)N_{S4}(t)dt = \alpha Rc_1V \times Rc_2(U+c_1V)G \times \int_0^{\infty}T(t)F(t)dt \tag{16}$$

Calculating effective R

The effective reproduction number, R^* is calculated as the ratio between the expected number of secondary cases and the expected number of primary cases:

$$\begin{aligned}R^* =& \frac{N_{S1}+N_{S2}+N_{S3}+N_{S4}}{N_P} \qquad (17)\\ =& R\left(U\frac{\int_0^{\infty}T(t)\left([(1-p)F(t)+pF(t-w)]G+pH(t,w)\right)dt}{\int_0^{\infty}T(t)F(t)dt}+c_1c_2VG\right) \qquad (18)\end{aligned}$$

Determining level of adherence required for a 5-day window to remain optimal

Let p_w denote the proportion of the population who are active users when the app implements a w-day window, and let $R^*(w)$ denote the effective reproduction number given a notification window of w. Letting $p_5 = \epsilon p_2$,

$0 \leq \epsilon \leq 1$, we observe that the value of ϵ required for a 5-day window to be optimal (when $R^*(5) < R^*(2)$) is independent of p_2:

$$\int_0^\infty T(t)\left([(1-\epsilon p_2)F(t) + \epsilon p_2 F(t-5)]G + \epsilon p_2 H(t,5)\right) dt < \int_0^\infty T(t)\left([(1-p_2)F(t) + p_2 F(t-2)]G + p_2 H(t,2)\right) dt \tag{19}$$

$$\iff -\epsilon p_2 \int_0^\infty T(t)(G[F(t) - F(t-5)] - H(t,5)dt < -p_2 \int_0^\infty T(t)(G[F(t) - F(t-2)] - H(t,2)dt \tag{20}$$

$$\iff \epsilon \int_0^\infty T(t)\left(G[F(t) - F(t-5)] - H(t,5)\right) dt > \int_0^\infty T(t)\left(G[F(t) - F(t-2)] - H(t,2)\right) dt \tag{21}$$

For a given window w, $R^*(w)$ decreases as p_w increases if and only if $\int_0^\infty T(t)\left(G[F(t) - F(t-w)] - H(t,w)\right) dt > 0$. Otherwise, $R^*(w)$ **increases** with increasing active app use. These instances are not considered here, as the intended control measure increases transmission. Given that $R^*(w)$ decreases as active app use increases, a 5-day window is optimal given that ϵ satisfies:

$$\epsilon > \frac{\int_0^\infty T(t)(G[F(t) - F(t-2)] - H(t,2))dt}{\int_0^\infty T(t)(G[F(t) - F(t-5)] - H(t,5))dt} \tag{22}$$

A numerical simulation of the model

The model described above can also be simulated directly. This may be useful to account for factors that are not easy to incorporate within the analytic framework. We introduce the notation R_i and f_i to denote the baseline R parameter for each individual i, and f_i (F_i) denote the probability density function (cumulative density function) of the infectivity profile for each individual i. Further, let us subdivide primary cases by symptomatic status and vaccination status:

- N_{P1}: Primary cases who are asymptomatic and unvaccinated
- N_{P2}: Primary cases who are symptomatic and unvaccinated
- N_{P3}: Primary cases who are asymptomatic and vaccinated
- N_{P4}: Primary cases who are symptomatic and vaccinated

such that $N_P = N_{P1} + N_{P2} + N_{P3} + N_{P4}$. Finally, let $\underline{N_P}$ and $\underline{N_S}$ denote vectors containing the number of primary cases from each simulated base case and the number of secondary cases from each simulated base case respectively. Pseudocode of the algorithm to numerically simulate the model is given in Algorithm 1.

Algorithm 1 Numerical simulation of the model

for $i \in \{1, ..., N\}$ **do**
  $d \sim$ detection time distribution
  $N_{P1} \sim \text{Poisson}(aR_i \times AU \times F_i(d))$
  $N_{P2} \sim \text{Poisson}(aR_i \times (1-A)U \times F_i(d))$
  $N_{P3} \sim \text{Poisson}(aR_i \times AVc_1 \times F_i(d))$
  $N_{P4} \sim \text{Poisson}(aR_i \times (1-A)Vc_1 \times F_i(d))$
  $N_P \leftarrow N_{P1} + N_{P2} + N_{P3} + N_{P4}$
  $\underline{N_P}(i) \leftarrow N_P$
  $N_{S1} = N_{S2} = N_{S3} = N_{S4} = 0$
  if $N_{P1} > 0$ **then**
    for $j \in \{1, ..., N_{P1}\}$ **do**
      $t \sim$ i's infectivity profile until day d
      if $(d - t) \leq w$ **and** random number $< p$ **then**
        $N_{S3} \leftarrow N_{S3} + \text{Poisson}(aR_j \times (U + c_1V) \times (F_j(d + l - t) + (1 - F_j(i_{notif}))))$
      else if random number $\geq p$ **then**
        $N_{S2} \leftarrow N_{S2} + \text{Poisson}(aR_j \times (U + c_1V))$
      else
        $N_{S1} \leftarrow N_{S1} + \text{Poisson}(aR_j \times (U + c_1V))$
      end if
    end for
  end if
  if $N_{P2} > 0$ **then**
    for $j \in \{1, ..., N_{P2}\}$ **do**
      $t \sim$ i's infectivity profile until day d
      $\tau \sim$ time to symptom onset distribution
      if $(d - t) \leq w$ **and** random number $< p$ **then**
        if $\tau < d + l - t$ **then**
          $N_{S3} \leftarrow N_{S3} + \text{Poisson}(R_j \times (U + c_1V) \times (F_j(\tau) + (1 - F_j(\tau + i_{sympt}))))$
        else if $\tau < i_{notif}$ **then**
          $N_{S3} \leftarrow N_{S3} + \text{Poisson}(R_j \times (U + c_1V) \times (F_j(d + l - t) + (1 - F_j(\tau + i_{sympt}))))$
        else
          $N_{S3} \leftarrow N_{S3} + \text{Poisson}(R_j \times (U + c_1V) \times (F_j(d + l - t) + (F_j(\tau) - F_j(i_{notif})) + (1 - F_j(\tau + i_{sympt}))))$
        end if
      else if random number $\geq p$ **then**
        $N_{S2} \leftarrow N_{S2} + \text{Poisson}(R_j \times (U + c_1V) \times (F_j(\tau) + (1 - F_j(\tau + i_{sympt}))))$
      else
        $N_{S1} \leftarrow N_{S1} + \text{Poisson}(R_j \times (U + c_1V) \times (F_j(\tau) + (1 - F_j(\tau + i_{sympt}))))$
      end if
    end for
  end if
  if $N_{P3} > 0$ **then**
    for $j \in \{1, ..., N_{P3}\}$ **do**
      $N_{S4} \leftarrow N_{S4} + \text{Poisson}(c_2 \times aR_j \times (U + c_1V))$
    end for
  end if
  if $N_{P4} > 0$ **then**
    for $j \in \{1, ..., N_{P4}\}$ **do**
      $\tau \sim$ time to symptom onset distribution
      $N_{S4} \leftarrow N_{S4} + \text{Poisson}(c_2 \times R_j \times (U + c_1V) \times (F_j(\tau) + (1 - F_j(\tau + i_{sympt}))))$
    end for
  end if
  $N_S \leftarrow N_{S1} + N_{S2} + N_{S3} + N_{S4}$
  $\underline{N_S}(i) \leftarrow N_S$
end for
  return $\underline{N_P}, \underline{N_S}$

Supplementary Note 1 Assuming the base case seeks a PCR test

This section considers a scenario where base cases are detected through a PCR test, rather than through an LFT. While LFTs can produce a result in 30 minutes, PCR tests must be processed in a laboratory. Accordingly, there is a delay in individuals receiving a result, with consequential delays in their potential infectious contacts being notified. Here, we assume a delay of two days. In Supplementary Figure 1, we observe qualitatively similar results regarding the impact of notification window length and active app use to those obtained assuming no delay.

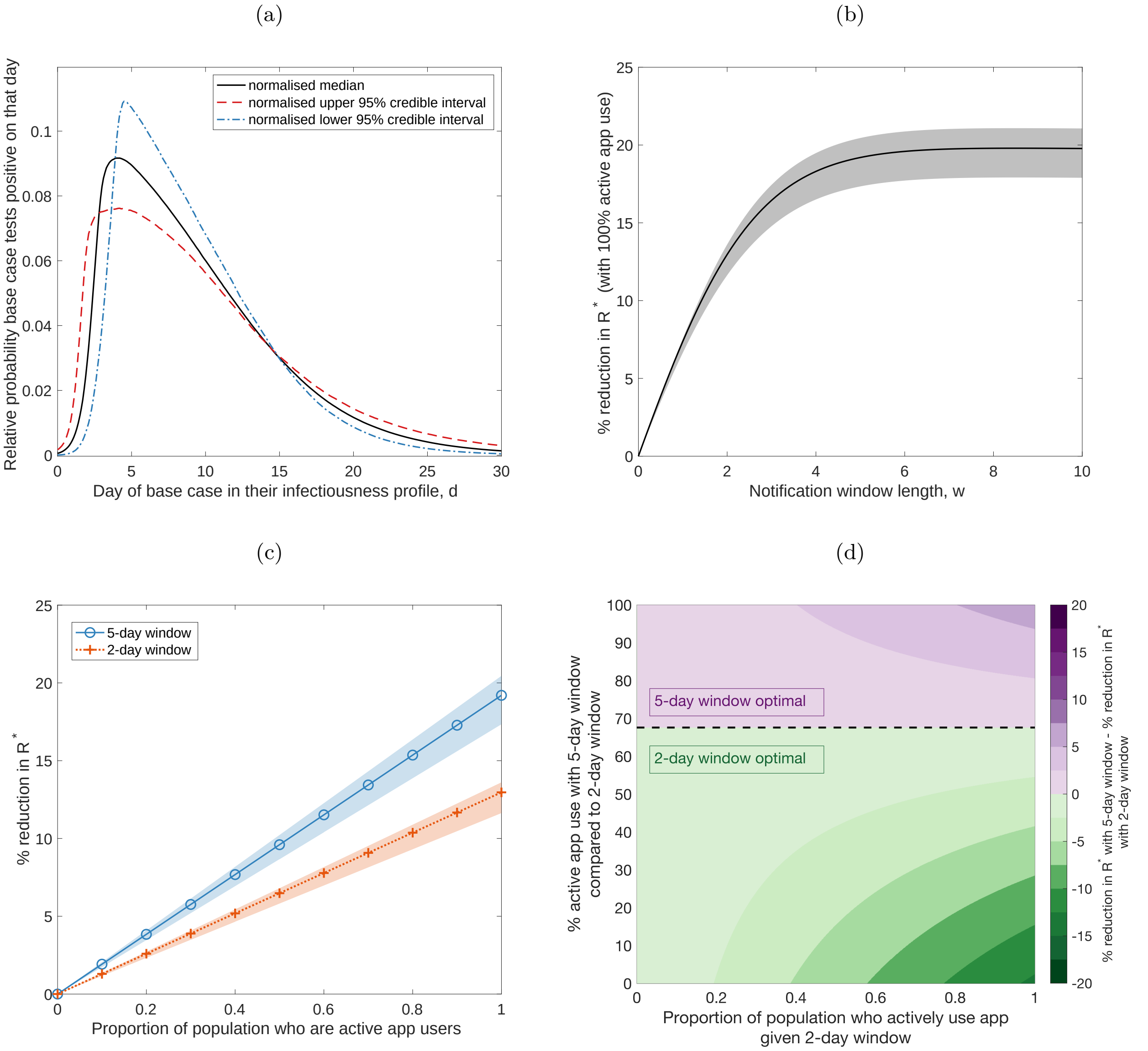

Supplementary Figure 1: **Impact of the notification window length and active app use on transmission, assuming the base case is detected through a PCR test.** (a) The relative probability of the base case testing positive on a given day in their infectiousness profile, obtained by normalising the median (black, solid line) test probability profiles of PCR tests for asymptomatic testing[1]. Normalised 95% credible interval test probability profiles (upper - red, dashed line; lower - blue, dot-dashed) are used to obtain shaded regions for (b) and (c). (b) The percentage reduction in R^* with respect to increasing length of the notification window w, under the assumption that all notified individuals adhere to isolation upon notification. (c) The relationship between probability of adherence and percentage reduction in R^* for 5-day notification windows (blue solid line, circle markers) and 2-day notification windows (orange dotted line, cross markers). (d) Difference in the percentage reduction in R^* that results from a 5-day notification window compared to a 2-day notification window. The proportion of primary cases assumed to be active app users for a 2-day window is shown on the x-axis, and the relative level of active app use assumed for a 5-day window (compared to the level of active app use for a 2-day window) is shown on the y-axis. Purple (green) regions correspond to where 5-day (2-day) notification windows lead to a larger reduction in R^*.

R^* is reduced by a lower extent in this instance for two reasons. Firstly, there is a delay between the base case taking a test and primary cases who they have infected being notified. Because of this, primary case infectious individuals can have a longer period to make contacts with others, with the potential for onward transmission of infection, before isolating. Secondly, owing to the greater sensitivity of PCR tests, individuals are more likely to be detected late in their infectious period. For late detected individuals, the majority of primary cases would be expected to have been infected prior to the notification window. Consequently, in this instance the transmission reduction from app-based notifications is considerably lower, with 2-day and 5-day notification windows resulting in a 13% and 19% reduction in transmission assuming an entire population of active app users respectively.

The results in Figure 2 and Supplementary Figure 1 are predicated on the assumption that a base case individual tests positive to an LFT or a PCR respectively. However, LFTs are less sensitive than PCR tests [14]. Because of this, our results should not be taken as a quantification of the relative merits of LFT and PCR testing. Rather, our results should be interpreted as: if an individual is detected by an LFT and can input their result into the app immediately, contact tracing will lead to a greater reduction in transmission than if they were detected by a PCR and must wait until the PCR result has returned. It is important to note that detection via PCR tests still contributes positively to contact tracing, particularly if PCR tests detect individuals who test negative to an LFT.

Supplementary Note 2 Assuming the base case takes a test upon symptom onset

This section considers a scenario where base cases are symptomatic and detected upon symptom onset, rather than being an asymptomatic and detected via an LFT (Main analysis) or via a PCR test (Supplementary Note 1). Using the incubation period distribution from the main analysis[11] as the time-detection distribution for symptomatic individuals (Supplementary Figure 2a), symptomatic individuals are more likely to be detected earlier in their infectiousness profile than an asymptomatic individual taking a test on a random day in their infectious period. Consequently, the reduction in R^* from detecting a symptomatic base at symptom onset is larger than the reduction in R^* from an asymptomatic base case, for both LFTs and PCR tests, at all notification window lengths (Supplementary Figure 2b). In all cases, there is limited further reductions to R^* for notification windows longer than five days.

(a) (b)

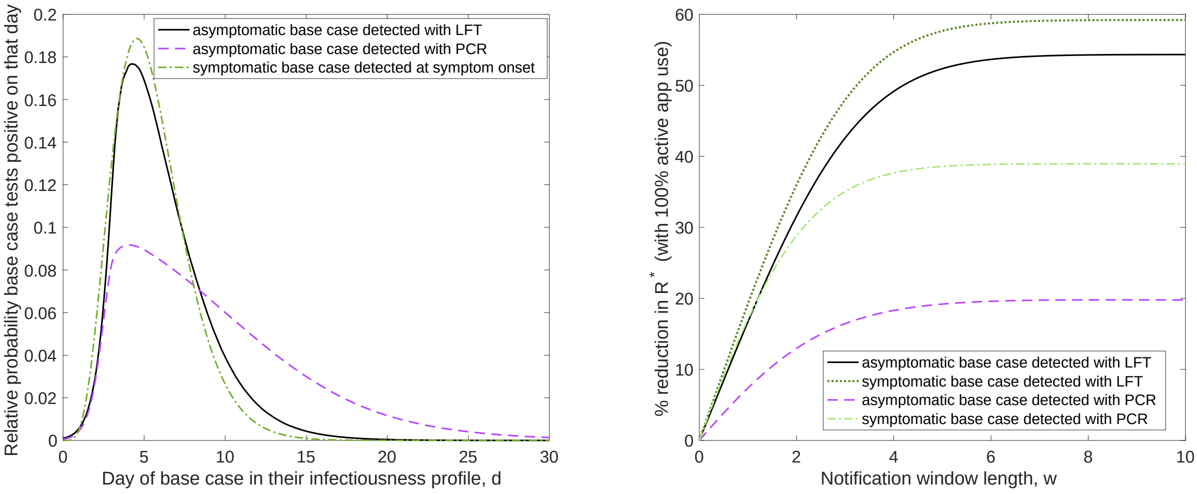

Supplementary Figure 2: **Impact of the notification window length on transmission, assuming a symptomatic base case.** (a) The relative probability of the base case testing positive on a given day in their infectiousness profile. For symptomatic base cases, we use the incubation period distribution[11] (green, dot-dashed line) as the test probability profile of both LFTs and PCR tests, while for asymptomatic the profiles for LFTs (black, solid line) and PCR tests (purple, dashed line) are obtained by normalising the median (black, solid line) test probability profiles of LFT tests for asymptomatic testing[1]. (b) The percentage reduction in R^* with respect to increasing length of the notification window w, under the assumption that all notified individuals adhere to isolation upon notification: for an asymptomatic base case detected via an LFT (black, solid line); for an asymptomatic base case detected via a PCR test (purple, dashed line); for a symptomatic base case detected via an LFT (dark green, dotted line); and for a symptomatic base case detected via a PCR test (light green, dot-dashed line).

Supplementary Note 3 Exploring the impact of the day of base case detection

Assuming that not all individuals are active app users, the reduction in transmission from the app depends upon: the day in which a base case individual is detected, d; on the number of their contacts notified (determined by the length of notification window, w); and on the proportion p of the population who are active app users, who adhere to self-isolation upon notification (Supplementary Figures 3a and 3b). For $p < 0.1$, neither of the considered notification window lengths reduces R^* by more than 10%. There is no difference in the reduction in R^* when $d < 2$ because all infectious contacts are captured by both notification windows. However, a 2-day notification window can be considerably less effective when base case individuals are detected at a later stage of their infection (Supplementary Figure 3c). The greatest difference in R^* occurs when the base case is detected between 7 and 8 days into their infectiousness profile, with the difference in R^* increasing with probability of adherence.

However, assuming that fewer individuals are likely to be active app users for a 5-day window than for a 2-day window, a 2-day window can become optimal for some regions of (d, p) space (Supplementary Figures 3d to 3f). Assuming that active app use given a 5-day notification window is 80%, 60%, and 40% of the active app use given a 2-day notification window, a 2-day notification window is optimal when $d < 4.8$ days, $d < 6.1$ days and $d < 7.9$ days respectively, with the greatest differences occurring when assuming a high level of active app use and very early detection of the base case individual.

Assuming the base case individual tests positive on day d from a one-off test (Supplementary Figure 4a), and assuming a proportion p of primary cases are active app users, the relative level of active app use required given a 5-day window to be optimal is independent of p (Supplementary Figure 4b, solid black line). If the base case tests positive early in their infectiousness profile, high relative levels of active app use are required for a 5-day notification window to be beneficial, while if a base case is detected late, a 5-day window may remain optimal despite the lower level of active app use. Because of this, it becomes increasingly important to identify asymptomatic cases early in their infection profiles for shorter notification windows. In our main analyses, we consider a base case individual who tests positive on a random day in their infectiousness profile. Here, we consider an alternative situation where the base case individual takes regular tests every r days. The probability an individual tests positive on a given day d when $d < r$ can be obtained directly from a test probability profile, while the probability an individual tests positive on day d when $d > r$ is the probability that individual tests positive on day d and did not test positive on subsequent test days, which can be inferred from a test probability profile. Individuals are more likely to be identified early in their infectious period if they engage in regular mass testing (Supplementary Figure 4a). Consequently, the required level of active app use given a 5-day window for that strategy to remain optimal increases. As an example, assuming a base case takes a LFT every seven days, a 2-day window is optimal given that active app use with a 5-day window is at least 62% of the level of active app use given a 2-day window (Supplementary Figure 4b, purple square); assuming a base case takes a lateral flow test every three days, a 5-day window is optimal given that active app use is at least 70% of the level of active app use given a 2-day window (Supplementary Figure 4b, pink diamond).

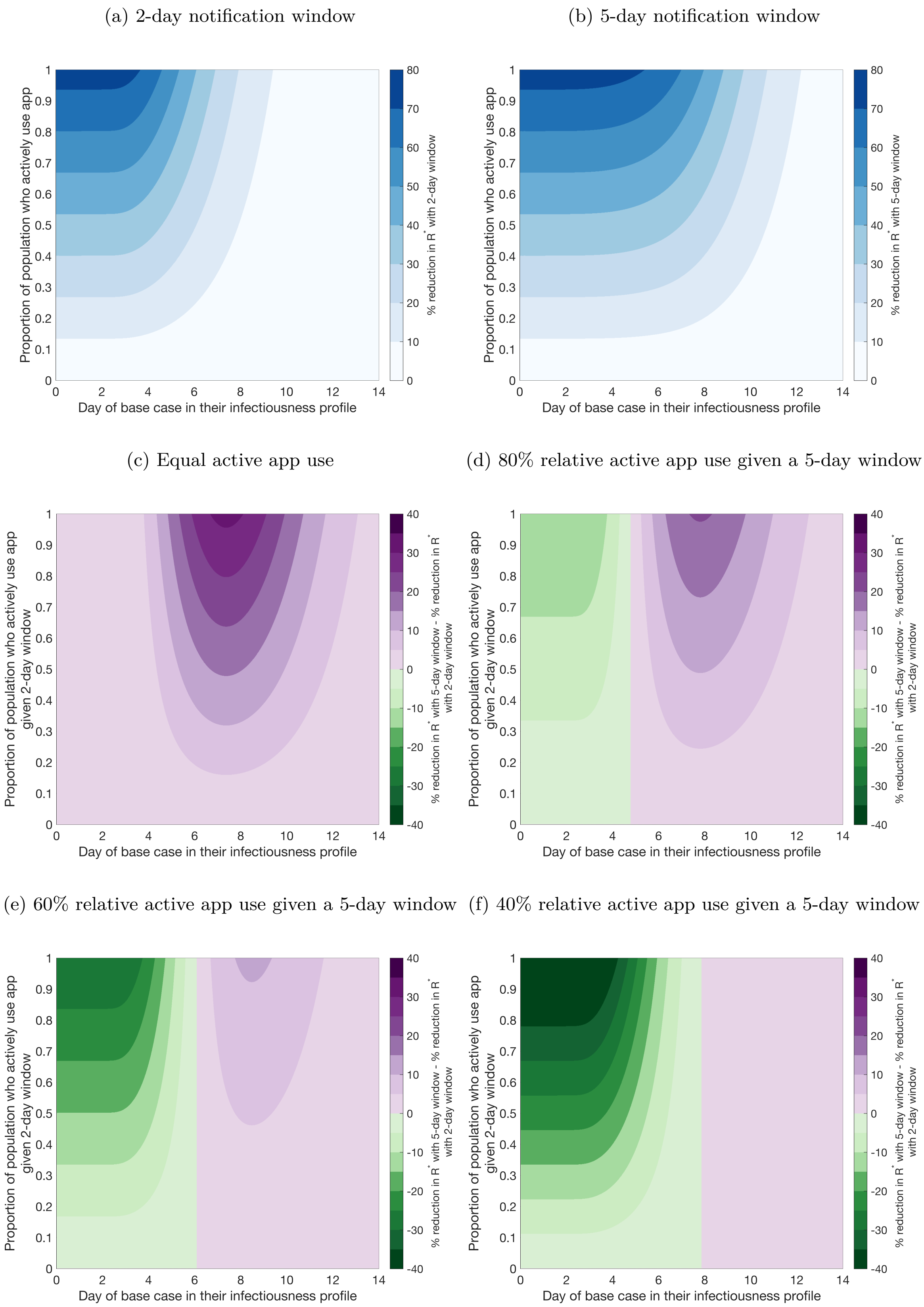

Supplementary Figure 3: **Impact of the detection time of the base case and active app use on transmission reduction.** (a & b) The percentage reduction in R^* that results from a 2-day and 5-day notification window, respectively. Darker shading corresponds to greater percentage reductions in R^*. (c-f) The difference in the percentage reduction in R^* that results from a 5-day and 2-day notification window, respectively, assuming that: (c) the proportion of the population who are active app users given $w = 2$ is equal to the proportion of the population who are active app users given $w = 5$, (d-f) the proportion of the population who are active app users given $w = 5$ is (d) 80%, (e) 60%, and (f) 40% of the proportion of the population who are active app users given $w = 2$.

(a) (b)

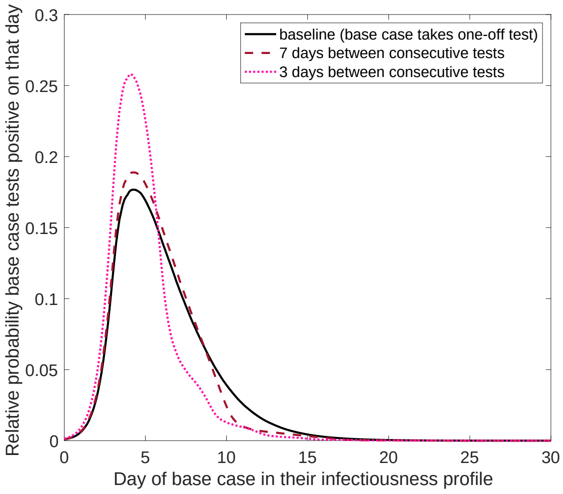

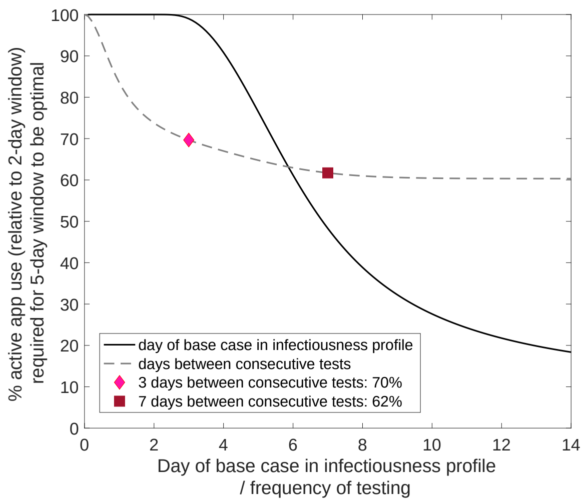

Supplementary Figure 4: **Impact of regular testing on the level of active app use required for a 5-day window to be optimal:** (a) The relative probability of a base case testing positive on a given day in their infectiousness profile, assuming they take a one-off test (black, solid line), assuming they take a test every seven days (dark red, dashed line), and assuming they take a test every three days (pink, dotted line). (b) The percentage active app use relative to the level of active app use given a 2-day window required for a 5-day notification window to result in a lower R^* when: the base case is detected on day d (black solid line); the base case takes a test every d days (grey dashed line). We highlight two examples: assuming a base case takes a lateral flow test every seven days, a 5-day window is optimal given that active app use is at least 62% of the level of active app use given a 2-day window (purple square); assuming a base case takes a lateral flow test every three days, a 5-day window is optimal given that active app use is at least 70% of the level of active app use given a 2-day window (pink diamond).

Supplementary Note 4 Exploring the impact of vaccine effectiveness

While vaccination may reduce both susceptibility to infection (c_1) as well as transmissibility upon infection (c_2), in the Supplementary Methods we observe that R^* is only impacted by their product, $c_1 \times c_2$. We refer to $(1 - c_1 \times c_2)$ as a vaccine's *effectiveness*. Here, we explore the impact of vaccine effectiveness on the effectiveness of app-based notifications at 2-day and 5-day windows at reducing R^*. Under our baseline assumptions $c_1 = 0.37$, $c_2 = 0.37$ we obtain a vaccine effectiveness of $\approx 86\%$. In Supplementary Figure 5, we observe higher levels of vaccine effectiveness result in larger reductions in R^*, because vaccinated individuals are not required to isolate upon notification. Hence, if vaccines are less effective, such individuals would be expected to infect a higher number of secondary cases.

(a)

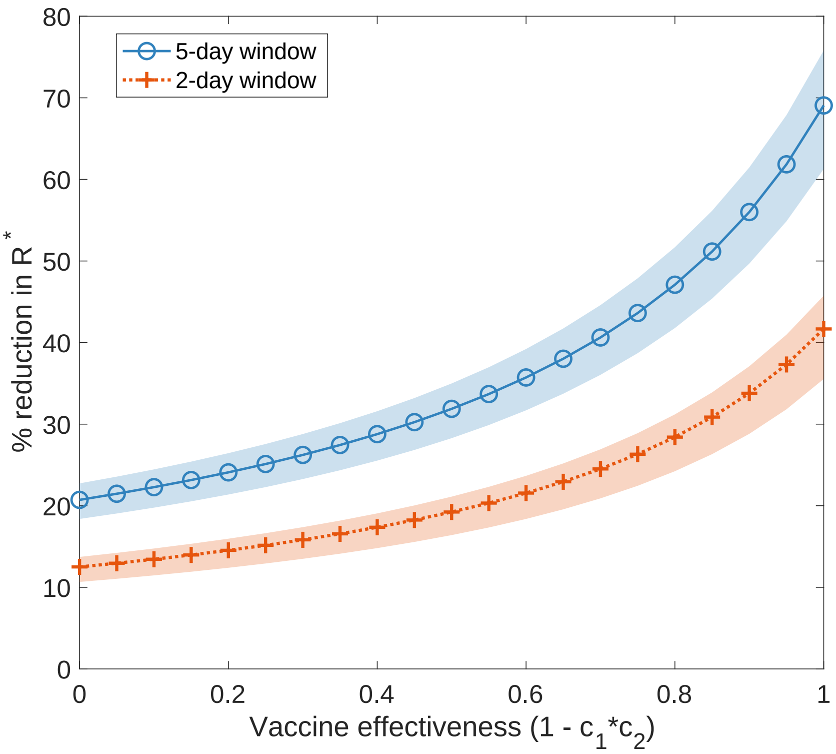

Supplementary Figure 5: **Impact of vaccine effectiveness on transmission reduction.** The percentage reduction in R^* with respect to vaccine effectiveness, defined as the product of the vaccine's impact on susceptibility (c_1) and transmissibility (c_2), for a 2-day window (orange dotted line, cross markers) and a 5-day window (blue solid line, circle markers), under the assumption that 70% of the population are fully vaccinated. Normalised 95% credible interval test probability profiles[1], used as the distribution of detection times of base cases, were used to obtain shaded regions.

Supplementary Note 5 Exploring the impact of heterogeneity

While our results are based on one infectiousness profile, this can be interpreted as the 'average' infectiousness profile of individuals within the population, and hence the method captures heterogeneity between individuals, as long as the generation time distribution of the population at large matches this infectiousness profile. Through numerical simulation, we demonstrate this for two types of heterogeneity.

Firstly, to consider the impact of heterogeneous contact rates between individuals, and the potential for some individuals to be superspreaders, we explored the impact of allowing the expected number of onward cases from base cases and primary cases to differ between individuals, while keeping the infectiousness profile of individuals through time the same for each individual. Specifically for each individual i, we draw a heterogeneity parameter $h_i \sim \text{LogNormal}(0, 1.1)$. R_i values were obtained by multiplying R by h_i, which is then rescaled to not increase the mean value of R_i over the population, i.e. we took $R_i = h_i \frac{R}{\mathbb{E}(h_i)}$. We chose this distribution to satisfy 80% of primary cases resulting from contact with 20% of base cases (Supplementary Figure 6a), broadly in line with studies of SARS-CoV-2 superspreading events from contact tracing data[15,16]. However, this distribution was chosen as an illustrative example rather than a precise distribution of contact rates or superspreading for SARS-CoV-2, and we emphasise that we would expect our results to hold irrespective of this choice of distribution. Despite the heterogeneity in contact rates, stochastic simulations closely match the results obtained from the analytic model (Supplementary Figures 6b and 6c).

Secondly, we explored the impact of heterogeneity in infectious periods - individuals were assumed to have a constant level of infectiousness throughout their period of infection, and infectious periods were drawn to match the infectiousness profile at a population level. We achieved this by drawing a parameter $k_i \sim \text{Unif}(0, \max_x f(x))$. The individual's infectious period is then taken to be the values of t such that $f(t) - k > 0$. Specifically, in order to satisfy $\frac{\sum_{i=1}^{N} f_i(t)}{N} \approx f(t)$, for each individual i we set $f_i(t)$ to be:

$$f_i(t) = \begin{cases} \max_x f(x), \text{ if } f(t) - k > 0 \\ 0, \text{ otherwise} \end{cases} \tag{23}$$

Supplementary Figures 7a and 7b demonstrate the sampling method and construction of infectious periods. This method is an illustrative example, chosen to satisfy the infectiousness profile at the population level. It should not be taken as an accurate distribution of infectiousness periods or times to onset of infectiousness. For example, by construction, the earlier a sampled infectious individual becomes infectious, the longer they remain infectious, which would not be a feature desired in a realistic model. For those wanting to accurately model heterogeneity in infectious periods, one would have to model both a time from exposure to infectiousness distribution and an infectious period distribution, for example by using the approach outlined by Hart et al.[2]. Again, we see that despite the heterogeneity in infectious periods between individuals (Supplementary Figure 7c), stochastic simulations closely match results from the analytic model (Supplementary Figures 7d and 7e).

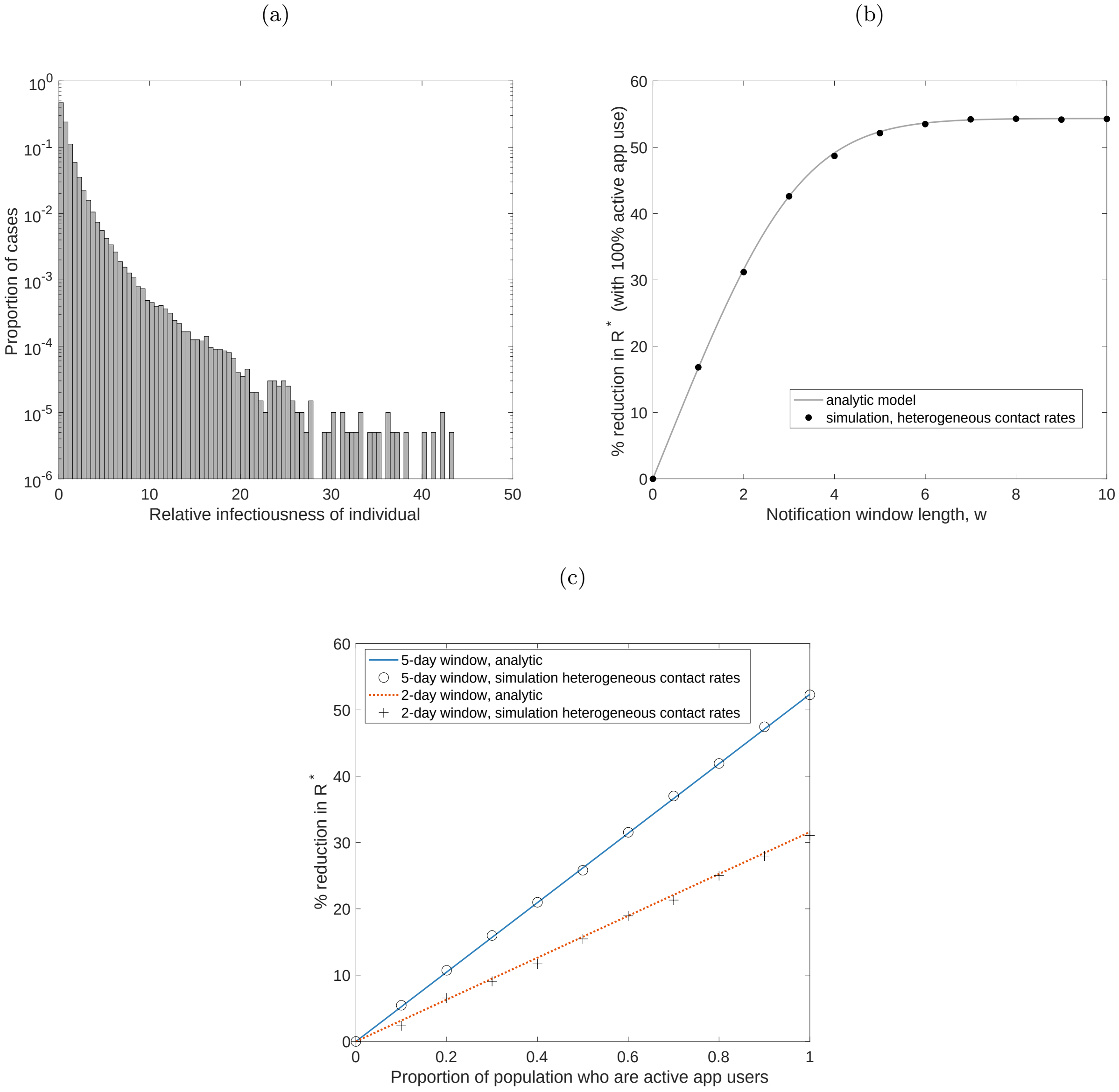

Supplementary Figure 6: **Exploring the impact of heterogeneity in infectiousness between individuals.** (a) A histogram of the relative infectiousness, h_i, of simulated individuals, in bins of width 0.5, with the y-axis plotted on a log scale, with $h_i \sim \text{LogNormal}(0, 1.1)/\mathbb{E}(h_i)$. (d) The percentage reduction in R^* for different length notification window w, relative to a scenario in which a notification app is not used, assuming all individuals are active app users, obtained from the analytic model (grey solid line) and via simulation of individuals with heterogeneous infectious periods (black markers). (c) The relationship between the proportion of primary cases who are active app users and percentage reduction in R^* from the analytic model for a 5-day window (blue solid line) and 2-day window (orange dotted line), and from simulation of individuals with heterogeneous infectious periods for a 5-day window (black circle markers) and 2-day windows (black cross markers). Values plotted for simulations are mean values from numerical simulation of 200,000 base cases.

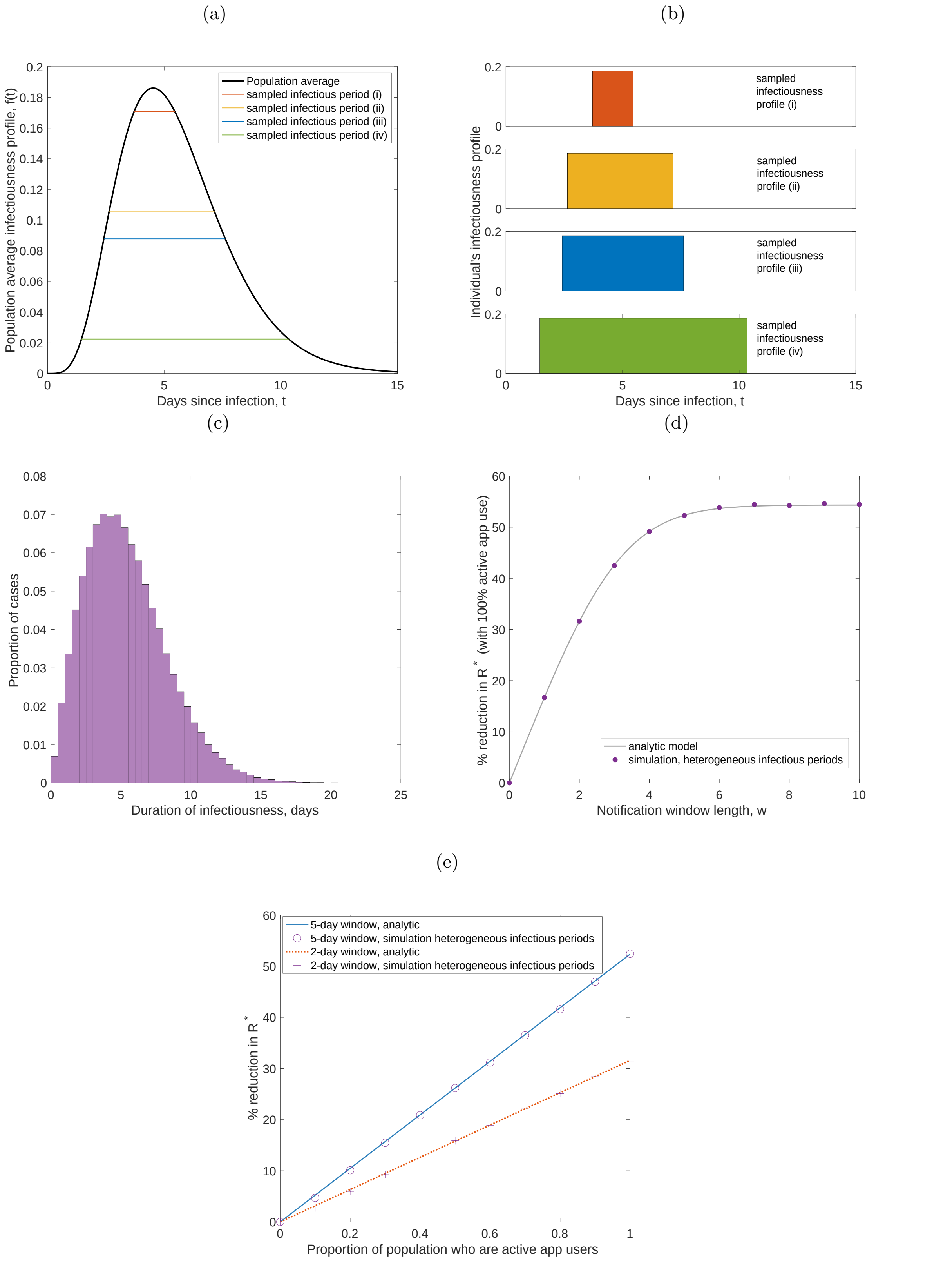

Supplementary Figure 7: **Exploring the impact of heterogeneity in infectious periods.** (a) illustrates the sampling method used to obtain simulated infectious periods - a random point $k \in (0, \max f(x))$ along the y-axis is drawn, with the infectious period taken as the values of t satisfying $f(t) - k > 0$. (b) Across infected individuals, a depiction of them having equal infectiousness (whilst infected) but variable infectious periods. (c) Duration of infectiousness of simulated individuals, in bins of width 0.5 days. (d) The percentage reduction in R^* for different length notification window w and assuming all individuals are active app users, relative to a scenario in which a notification app is not used. Obtained from the analytic model (grey solid line) and via simulation of individuals with heterogeneous infectious periods (purple markers). (e) The relationship between the proportion of primary cases who are active app users and percentage reduction in R^* from the analytic model for a 5-day window (blue solid line) and 2-day window (orange dotted line), and from simulation of individuals with heterogeneous infectious periods for a 5-day window (purple circle markers) and 2-day windows (purple cross markers). Values plotted for simulations are mean values from numerical simulation of 200,000 base cases.

Supplementary References

[1] Hellewell J, Russell TW, Beale R, Kelly G, Houlihan C, Nastouli E, et al. Estimating the effectiveness of routine asymptomatic PCR testing at different frequencies for the detection of SARS-CoV-2 infections. BMC Medicine. 2021;19:106. Available from: https://bmcmedicine.biomedcentral.com/articles/10.1186/s12916-021-01982-x.

[2] Hart WS, Maini PK, Thompson RN. High infectiousness immediately before COVID-19 symptom onset highlights the importance of continued contact tracing. Elife. 2021;10:e65534.

[3] Chadeau-Hyam M, Wang H, Eales O, Haw D, Bodinier B, Whitaker M, et al. SARS-CoV-2 infection and vaccine effectiveness in England (REACT-1): a series of cross-sectional random community surveys. The Lancet Respiratory Medicine. 2022.

[4] de Gier B, Andeweg S, Backer JA, Hahné SJ, van den Hof S, de Melker HE, et al. Vaccine effectiveness against SARS-CoV-2 transmission to household contacts during dominance of Delta variant (B. 1.617. 2), the Netherlands, August to September 2021. Eurosurveillance. 2021;26(44):2100977.

[5] UK Government. Coronavirus (COVID-19) in the UK dashboard: Vaccination uptake, by report date; 2021. Available from: https://coronavirus.data.gov.uk/details/vaccinations.

[6] UK Government. Press release: NHS COVID-19 app updated to notify fewer contacts to isolate; 2021. Available from: https://www.gov.uk/government/news/nhs-covid-19-app-updated-to-notify-fewer-contacts-to-isolate.

[7] UK Government. Guidance for contacts of people with confirmed coronavirus (COVID-19) infection who do not live with the person; 2021. Available from: https://www.gov.uk/government/publications/guidance-for-contacts-of-people-with-possible-or-confirmed-coronavirus-covid-19-infection-who-do-not-live-with-the-pe

[8] Alene M, Yismaw L, Assemie MA, Ketema DB, Mengist B, Kassie B, et al. Magnitude of asymptomatic COVID-19 cases throughout the course of infection: A systematic review and meta-analysis. PloS one. 2021;16(3):e0249090.

[9] McEvoy D, McAloon C, Collins A, Hunt K, Butler F, Byrne A, et al. Relative infectiousness of asymptomatic SARS-CoV-2 infected persons compared with symptomatic individuals: a rapid scoping review. BMJ open. 2021;11(5):e042354.

[10] UK Government. Stay at home: guidance for households with possible or confirmed coronavirus (COVID-19) infection; 2021. Available from: https://www.gov.uk/government/publications/guidance-for-contacts-of-people-with-possible-or-confirmed-coronavirus-covid-19-infection-who-do-not-live-with-the-pe

[11] Lauer SA, Grantz KH, Bi Q, Jones FK, Zheng Q, Meredith HR, et al. The Incubation Period of Coronavirus Disease 2019 (COVID-19) From Publicly Reported Confirmed Cases: Estimation and Application. Annals of Internal Medicine. 2020 3.

[12] Ferretti L, Wymant C, Kendall M, Zhao L, Nurtay A, Abeler-Dörner L, et al. Quantifying SARS-CoV-2 transmission suggests epidemic control with digital contact tracing. Science. 2020 may;368(6491):eabb6936. Available from: https://www.sciencemag.org/lookup/doi/10.1126/science.abb6936.

[13] Deng Y, You C, Liu Y, Qin J, Zhou XH. Estimation of incubation period and generation time based on observed length-biased epidemic cohort with censoring for COVID-19 outbreak in China. Biometrics. 2020.

[14] Pickering S, Batra R, Merrick B, Snell LB, Nebbia G, Douthwaite S, et al. Comparative performance of SARS-CoV-2 lateral flow antigen tests and association with detection of infectious virus in clinical specimens: a single-centre laboratory evaluation study. The Lancet Microbe. 2021;2(9):e461-71.

[15] Adam DC, Wu P, Wong JY, Lau EH, Tsang TK, Cauchemez S, et al. Clustering and superspreading potential of SARS-CoV-2 infections in Hong Kong. Nature Medicine. 2020;26(11):1714-9.

[16] Endo A, et al. Estimating the overdispersion in COVID-19 transmission using outbreak sizes outside China. Wellcome open research. 2020;5.
